# Laccase-Based Biosensor Encapsulated in a Galactomannan-Chitosan Composite for the Evaluation of Phenolic Compounds

**DOI:** 10.3390/bios10060070

**Published:** 2020-06-22

**Authors:** Imane Boubezari, François Bessueille, Anne Bonhomme, Gaëtan Raimondi, Ali Zazoua, Abdelhamid Errachid, Nicole Jaffrezic-Renault

**Affiliations:** 1 Laboratory of Applied Energetics and Materials, University of Jijel, Ouled Aissa 18000, Algeria; boubezari.imen@gmail.com (I.B.); azazoua@yahoo.fr (A.Z.); 2 Institute of Analytical Sciences, University of Lyon, 69100 Villeurbanne, France; francois.bessueille@univ-lyon1.fr (F.B.); anne.bonhomme@isa-lyon.fr (A.B.); gaetan.raimondi@isa-lyon.fr (G.R.); abdelhamid.errachid-el-salhi@univ-lyon1.fr (A.E.)

**Keywords:** galactomannan, chitosan, laccase, voltammetry, phenolic content

## Abstract

Galactomannan, a neutral polysaccharide, was extracted from carob seeds and characterized. It was used for the first time for the fabrication of a laccase-based biosensor by the encapsulation of laccase in a chitosan+galactomannan composite. The fabricated biosensor was characterized by FTIR, scanning electron microscopy and cyclic voltammetry. The pyrocatechol detection was obtained by cyclic voltammetry measurements, through the detection of o-quinone at −0.447 V. The laccase activity was well preserved in the chitosan+galactomannan composite and the sensitivity of detection of pyrocatechol in the 10^−16^ M–10^−4^ M range was very high. The voltammetric response of the biosensor was stable for more than two weeks. To estimate the antioxidant capacity of olive oil samples, it was shown that the obtained laccase-based biosensor is a valuable alternative to the colorimetric Folin–Ciocalteu method.

## 1. Introduction

Galactomannans are linear polysaccharides based on a -(l-4)-mannane backbone to which single D-galactopyranosyl residues are attached via -(l-6) linkages. They can be extracted from different seeds (carob [1] and *Trigonella foenum-graceum* [2]). Their molecular composition depends on their origin, in particular the galactose content and the mannose/galactose ratio. The molecular size and the fine structure (mannose/galactose ratio and galactose distribution in the mannose linear chain) influence solubility, ability to self-associate and control the rheological properties of their aqueous solutions. Galactomannans are industrially used in many domains: paper, textile, pharmaceutical, cosmetic and in food products as E410 additive (ice cream and other preparations). The main important property of this biopolymer is its ability to form a very viscous solution at relatively low concentration, to stabilize dispersion and emulsion and to replace fat in many dairy products. In this paper galactomannan was extracted from carob seeds. Galactomannan was used in interpenetrating polymeric network with chitosan [3] and in polyvinyl alcohol [4] for the encapsulation of drugs in nanoparticles and their control release. This polysaccharide was used for the encapsulation of enzymes in delivery systems of antimicrobial peptides and enzymes (protease and lipase), their own activity being well preserved [5].

Due to their antioxidant character, phenolic compounds, secondary plant metabolites, have been attracting the attention of scientists in recent years. They can also protect against many human diseases such as cancer or cardiovascular disease [6]. For these reasons, an important effort has been made to characterize the phenol content in plant tissues [7]. The highly sensitive methods such as GC/MS or HPLC/MS are expensive, time consuming and need a pretreatment step of the sample. Electrochemical biosensors [8], based on the immobilization of an enzyme such as laccase or tyrosinase, constitute an excellent alternative.

Several laccase-based biosensors were fabricated through the encapsulation of laccase in chitosan including nanomaterials or chitosan+carrageenan, as presented in Table 1.

The advantages for the encapsulation of an enzyme such as laccase in a polysaccharide (chitosan) are that its activity is preserved and some interferences are canceled. The integration of nanomaterials in chitosan increases the sensitivity of detection. It was demonstrated that the use of a chitosan–carrageenan complex for the encapsulation of an enzyme such as glucose oxidase increases the sensitivity of the voltammetric detection of glucose [19].

In this work, a galactomannan/chitosan (CHIT) polymer composite was used for the encapsulation of the enzyme laccase in a voltammetric biosensor because it was previously shown that galactomannan was able to preserve enzyme activity and this property has never applied to date to a biosensor design. The enzyme/polymer composite was deposited on a thiol modified gold electrode and then cross-linked in a glutaraldehyde vapor, through the formation of imide links between glutaraldehyde and the amine groups of chitosan. The detection of catechol was obtained through the voltammetric detection of the 1,2-benzohydroquione, product of the enzymatic reaction. The analytical performance of the fabricated laccase-based biosensor was determined for catechol detection. The phenolic content of olive oil was tested on diluted olive oil samples, without any further preparation procedure. The phenolic content of olive oil (main compound: hydroxytyrosol) confers to this product antioxidant, cardioprotective, ROS scavenging and anticancer properties [20]. A comparison of olive oils from different origins was operated with the fabricated laccase-based voltammetric biosensor and with a common colorimetric test.

## 2. Materials and Methods

### 2.1. Reagents

Sulfuric acid, ethanol, isopropanol, acetone, acetic acid,4-aminothiophenol (4-ATP, 97%), chitosan (CHIT; average MW = 45 kDa with a degree of acetylation >75.0%), glutaraldehyde (GA), pyrocatechol (≥99%), laccase (Enzyme Commission number (EC) = 1.10.3.2, from *Trametes versicolor*, 85 0.94 U/mg), Folin and Ciocalteu’s phenol reagent (2 M) and sodium carbonate were purchased from Sigma-Aldrich. Aqueous solutions were prepared using Milli Q water (resistivity 18.2 MΩ∙cm). Phosphate buffer solution, PBS (0.1 M), was prepared from monopotassium and dipotassium phosphate salts, sodium and potassium chlorides and adjusted to pH 7.

### 2.2. Extraction of Galactomannan from Carob Seeds (Ceratonia Siliqua)

The experimental procedures were as follows [1]:

Carob seeds are composed of hull, endosperm and germ. They were separated from cloves, using pincers.

The hull was manually separated from the endosperm after heating the carob seeds in a sulfuric solution (H_2_SO_4_/H_2_O 60/40 v/v), at 60 °C for 1 h. This treatment carbonizes the hull. The germs were then manually separated by splitting lengthwise the endosperm, after soaking in water for one night. In order to obtain the raw carob gum (locust bean gum (LBG)), the endosperms were washed, dried and grinded. The LBG was dissolved in hot water (80 °C) for one night. The solution was then centrifuged and the supernatant was recovered. After addition of isopropanol at 40 °C, centrifugation, the base was recovered as the white gum that contains galactomannan (GAL). From the raw carob gum, the recovery rate was 13%. 

### 2.3. Analytical Characterization of LAC/CHIT + GAL

FTIR spectra were recorded on the galactomannan film and on the laccase/galactomannan+chitosan film drop-coated on the gold electrode, by using a Nicolet Continuum microscope coupled with Nexus infrared spectroscopy in the specular reflectance mode, equipped with an MCT detector. The resolution used for measurements was equal to 4 cm^−1^ and the signal was processed through Happgenzel apodization.

The average molar masses of galactomannan dissolved in water (1% (w/w) was determined by SEC chromatography, using an Agilent Technologies system [16].

Scanning electron microscopy (SEM) images were realized on the galactomannan film and on the laccase/galactomannan+chitosan film drop-coated on the gold electrode, by using a VEGA TESCAN SEM.

Cyclic voltammetry (CV) measurements were carried out at room temperature using a Bio-Logic SPS analyzer instrument (Bio-Logic, France). Data processing was realized via EC LAB software. A 5 mL three electrode cell equipped with a platinum plate as a counter electrode, an Ag/AgCl electrode as a reference one and a modified gold plate as a working electrode was used. For the electrochemical characterization of the modified gold electrode, the CV measurements were performed in 10 mM [Fe[(CN)_6_]^3−/4−^ in PBS (0.1 M, pH 7), at a scan rate of 80 mV/s. For the determination of the analytical performance of the biosensor, the CV measurements were performed in presence of different concentrations of pyrocatechol in PBS solution (0.1 M, pH 7), at a scan rate of 80 mV/s.

### 2.4. Preparation of the Laccase Modified Gold Electrode

**Figure 1:**
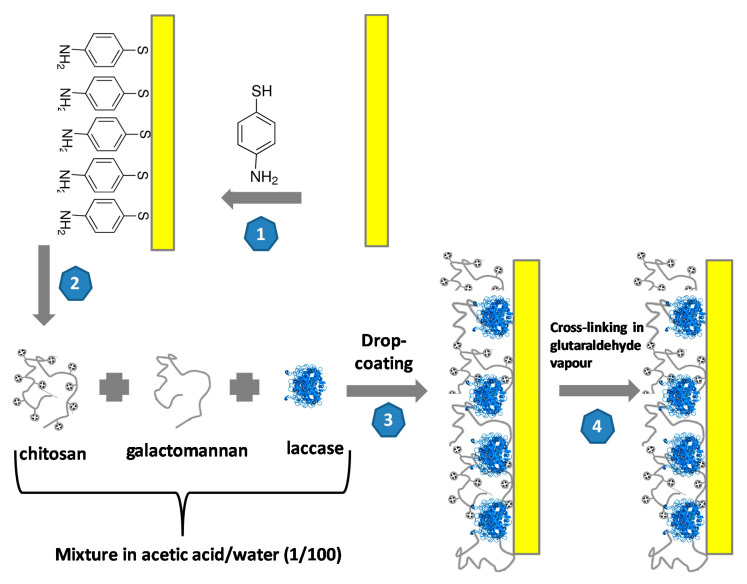
Schematic illustration the fabrication of the LAC/CHIT+GAL film on the gold electrode.

The gold substrates (300 nm of gold layer/30 nm of titanium layer/300 nm SiO2/p-type Si/300 nm SiO_2_) were supplied by the French RENATECH Network (LAAS, CNRS Toulouse, France). The surface of the electrode was cleaned in acetone for 5 min, then in UP water and finally incubated in a piranha solution (H_2_SO_4_:H_2_O_2_ = 3:1 v/v), then cleaned in UP water with drying under nitrogen flow. The schematic illustration the fabrication of the LAC/CHIT+GAL film on the gold electrode is presented in Figure 1.

Step 1: formation of the 4-ATP self-assembled monolayer; Step 2: formation of laccase/chitosan+galactomannan mixture; Step 3: Drop-coating of the laccase/galactomannan+chitosan film on the gold electrode and Step 4: Cross-linking in the glutaraldehyde vapor for 20 min.

The cleaned electrodes were incubated in 0.1 M ethanolic solution of 4-ATP for 12 h to form an anchored self-assembled monolayer (SAM) on the electrode surface. The electrode was then rinsed with ethanol.

A gel solution was prepared by mixing 0.5 mL of S1 solution and 0.5 mL of S2 solution:

- S1: 10 mg of chitosan dissolved in 0.1 mL of acetic acid + 4.9 mL of water after 24 h.
- S2: 10 mg of galactomannan dissolved in 5 mL of hot water (45 °C) after 5 h.

Ten milligrams of laccase was mixed in the gel solution. Ten microliters of this laccase/chitosan+galactomannan mixture was deposited on the 4-ATP/gold electrode surface. Then this surface was exposed to a glutaraldehyde vapor for an optimized duration time 20 min. The glutaraldehyde vapor was generated in a closed vessel, above a 25% glutaraldehyde solution in water, at ambient temperature. This vapor contained glutaraldehyde monomers that crosslinked amino groups from chitosan and from 4-ATP by forming imine groups, therefore this step insured the anchoring of the cross-linked laccase/chitosan+galactomannan film on the gold electrode and decreased the hydration rate of the film. Then LAC/CHIT+GAL modified gold electrode was kept at 4 °C for 24 hrs.

### 2.5. Application of the Biosensor for Detection of Phenolic Content in Olive Oil Samples

Olive oils obtained from different origins (Tunisia, Algeria, Morocco and Spain) were tested. Olive oils consist of mixtures refined olive oils and of virgin olive oils. Samples were stored in the dark at room temperature and opened just before use to prevent the oxidative degradation of the samples. Under optimized conditions, 5 mL of hexane standards or oil samples (1 g) diluted to 5 mL with hexane were placed in test tubes. Then, 100 μL of aqueous 1 M HCl solution were added and the mixture was shaken for 2 min using vortex agitation. Next, phases were separated by centrifugation for 10 min at 4000 rpm. The upper organic phase was carefully removed with a glass pipette and the remaining acidic aqueous phase (i.e., 40 μL) was retrieved with a syringe for final analysis. Cyclic voltammetric measurements were conducted in this extract, after dilution in PBS 0.1 M, pH 7 for the electrochemical detection of phenolic content. A colorimetric test was conducted in this extract by using the Folin–Ciocalteu reagent for comparative purposes. The Folin–Ciocalteu reagent was composed of a mixture of phosphotungstic acid H_3_PW_12_O_40_ and of phosphomolybdic acid H_3_PMo_12_O_40_ that are able to oxidize the phenolic compounds and then become reduced, leading to an increase of the absorption at 765 nm [21]. Forty microliters of the extract was mixed with 200 μL of the Folin–Ciocalteu reagent and 800 μL of 7.5% Na_2_CO_3_ solution, diluted up to 4 mL and incubated for 2 h in the dark before spectrophotometric determination; the absorbance was measured at 765 nm. The calibration curve was constructed using caffeic acid aqueous standards from 0 to 300 mg/L (*N* = 5) in 1 M HCl.

## 3. Results and Discussion

### 3.1. Characterization of the Prepared Galactomannan 

**Figure 2:**
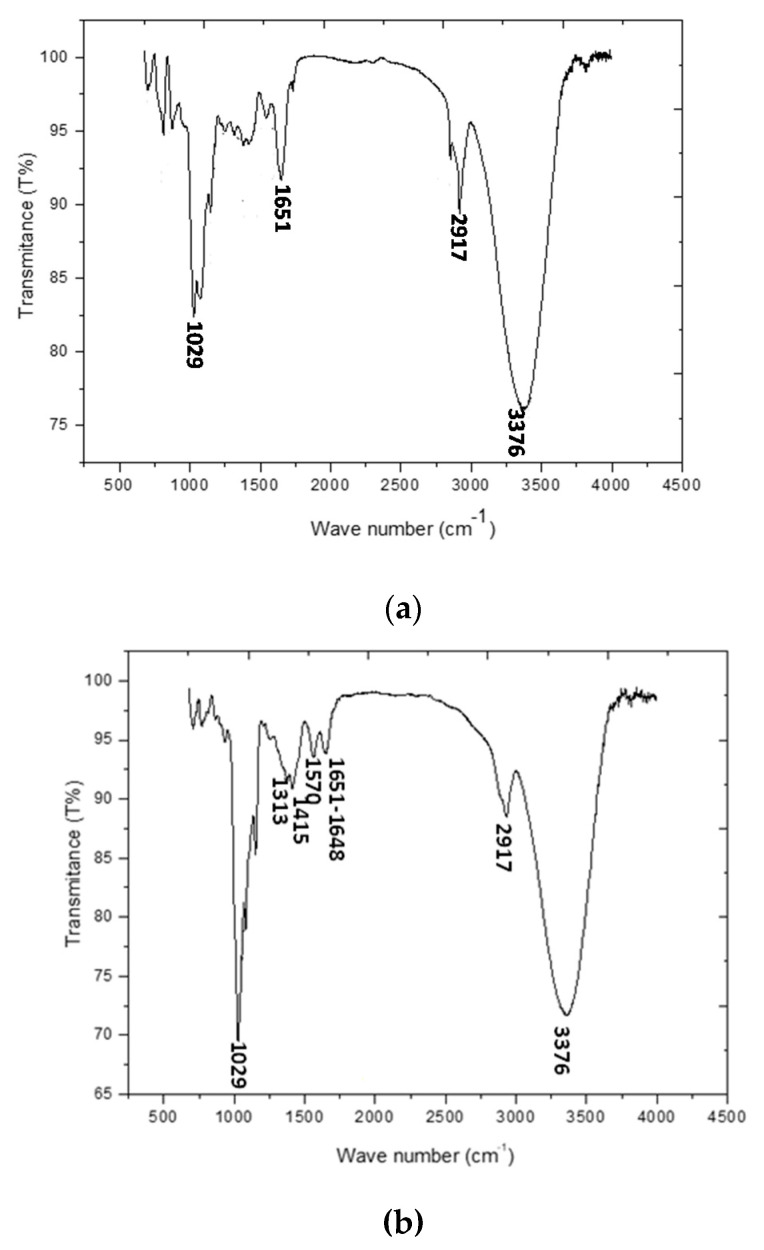
(**a**) FTIR spectrum of the prepared galactomannan and (**b**) FTIR spectra of the prepared laccase/galactomannan+chitosan film.

The FTIR spectrum of galactomannan is shown in Figure 2a. As they were previously identified [4], the characteristics absorption bands of galactomannan are present: the absorption band at 1651 cm^−1^ is of the ketonic carbonyl group. GAL showed characteristic peaks at 3376 cm^−1^ for O-H stretching of hydroxyl group and near about 2917 cm^−1^ For CH2 bending/wagging. A broad peak at 1029 cm^−1^ represented C-O-H stretching.

The molecular weight of galactomannan is found to be 850 kDa with a polydispersity index of 1.57. This value is close to that found for galactomannan extracted after acidic treatment of the carob seeds [1]. In this type of GAL, the mannose/galactose ratio was found to be 3.6, according to the molecular unit presented in Figure S1. This type of polysaccharide presents a non-ionic character [3], then it can be easily incorporated in a positively charge polymer such as chitosan. The global charge of this composite is then positive. The laccase whose isoelectric point is 3.6 [22] is negatively charged at pH 5-6 and can be tightly encapsulated in this positively charged composite.

### 3.2. Characterization of the Gold Electrode Modified with the Laccase/Chitosan+Galactomannan Film

• Spectroscopic characterization

FTIR spectrum of the prepared laccase/galactomannan+chitosan film is presented in Figure 2b. The main peaks of galactomannan (1651 cm^−1^, 3376 cm^−1^, 2917 cm^−1^ and 1029 cm^−1^) and of chitosan (1415 cm^−1^ and 1313 cm^−1^) are reported. The presence of the encapsulated laccase in the chitosan+galactomannan film was evidenced by the occurrence of two absorption bands at 1570 cm^−1^ and 1648 cm^−1^ characteristics of respectively amide I and amide II of laccase [23].

• Microscopic characterization

**Figure 3:**
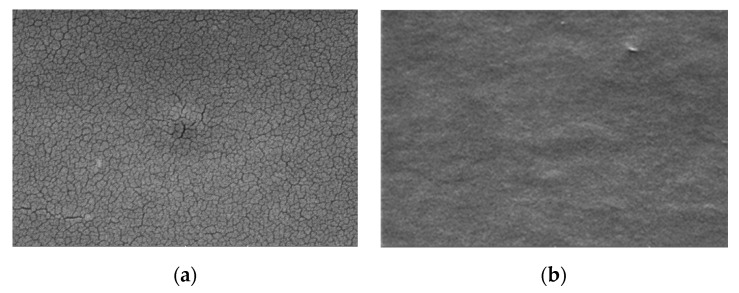
SEM images of the chitosan+galactomannan film without (**a**) and with the encapsulated laccase (**b**).

SEM images of the galactomannan+chitosan film without and with encapsulated laccase are presented respectively in Figure 3a,b. Galactomannan+chitosan film presents some cracks whereas the encapsulation of the hydrophilic laccase brings a more homogenous film with a globular structure.

• Electrochemical characterization

**Figure 4:**
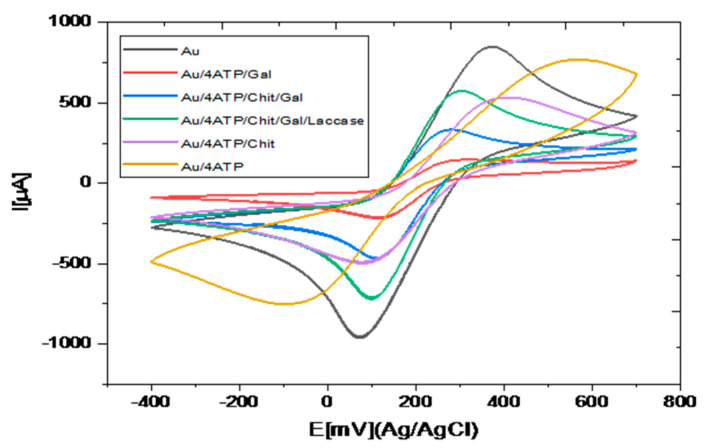
Cyclic voltammograms obtained for Au, Au/4-ATP, Au/4-ATP/CHIT, Au/4-ATP/GAL, Au/4-ATP/CHIT/GAL and Au/4-ATP/Chit/GAL/Laccase: 10 mM [Fe[(CN)_6_]^3−/4−^ in PBS (0.1 M, pH 7).

Cyclic voltammetric method, in the presence of the redox probe [Fe(CN)_6_]^3−/4−^, allows the study of the charge transfer at the modified electrode/electrolyte interface. The cyclic voltammograms obtained during the successive steps for the preparation of the modification of the gold electrode are presented in Figure 4.

Table S1 reported redox peak maximum intensities (Ia and Ic) and Ep and for the different steps of the modification of the gold electrode. A large increase of Ep is observed after the thiol SAM formation on the gold electrode surface. The high viscosity of the galactomannan film led to a large decrease of the peak maximum intensities; the insertion of galactomannan in chitosan led to a large increase of the peak maximum intensities by a factor higher than 2, showing the improvement of the charge transfer due to the presence of chitosan. This point shows the interest of using a galactomannan = chitosan composite rather than pure galactomannan for the electrochemical detection. After encapsulation of laccase, the peak maximum intensities are doubled, due to the presence of copper in the active site of the enzyme [24].

### 3.3. Analytical Performance of the Biosensor

Laccase catalyzes the oxidation of pyrocatechol to 1,2-benzoquinone according to the following equation:

$$\mathrm{catechol}+O_{2}\;\overset{Laccase}{\to }\;\mathrm{o-}\mathrm{quinone}+H_{2}O$$

o-quinone can be then electrochemically reduced to pyrocatechol at the surface of the proposed biosensor, as indicated by the electrochemical reaction:

o-quinone + 2H^+^ + 2e^−^ → catechol.

**Figure 5:**
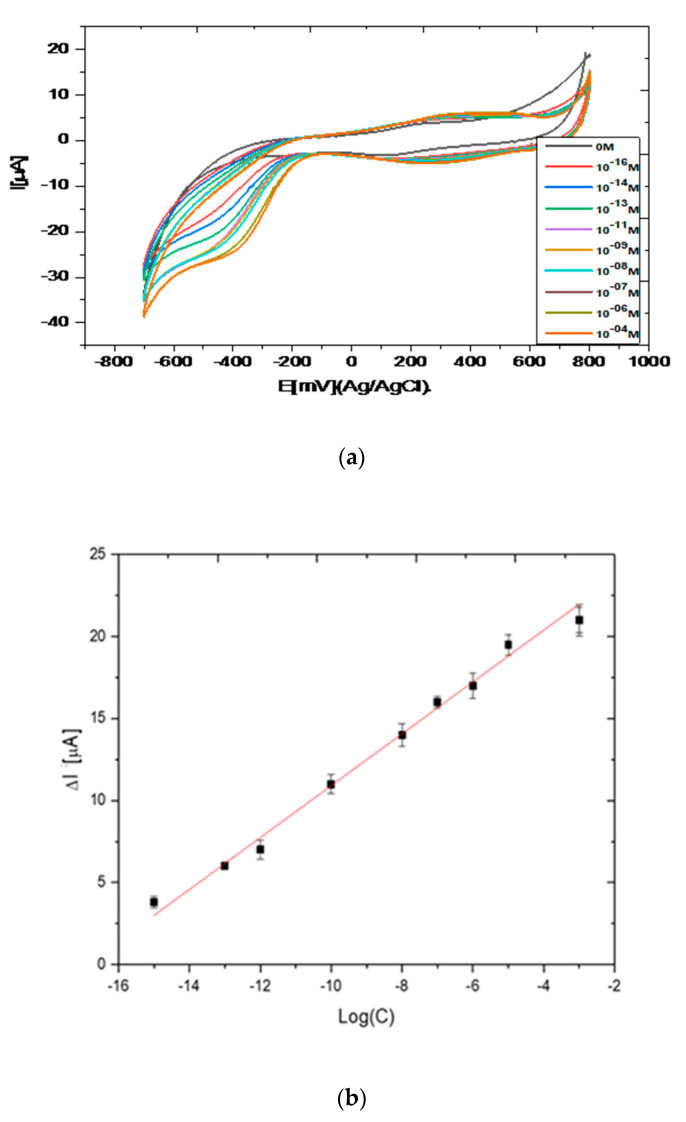
(**a**) Cyclic voltammograms observed for laccase/CHIT+GAL modified gold electrode in the presence of different concentrations of pyrocatechol. Scan rate of 80 mV/s. PBS solution (0.1 M, pH 7); (**b**) calibration curve of the laccase-based biosensor: variation of the peak maximum intensity versus logarithm of concentration of pyrocatechol.

In Figure 5a, cyclic voltammograms of the laccase/CHIT+GAL modified gold electrode in the presence of pyrocatechol are presented. The reduction peak corresponding to the reduction of o-quinone appeared at −447 mV for concentrations of pyrocatechol of 10^−16^ M. When concentrations of pyrocatechol increased, the potential of the reduction peak was shifted towards positive potentials until -380 mV for a pyrocatechol concentration of 10^−4^ M. The value of this cathodic potential was very low compared to that observed when laccase was encapsulated in chitosan + Carrageenan (−1.35 V) [16]. This point shows that the charge transfer was easier in CHIT+GAL film.

The intensity of the cathodic peak increased when the concentration of pyrocatechol increased. The variation of the intensity of the cathodic peak maximum was reported versus the concentration of pyrocatechol concentration, in the range 10^−16^–10^−4^ M in Figure 5a A linear relation was observed between the I (intensity for a concentration of pyrocatechol–intensity without catechol) and logarithm of the pyrocatechol concentration, in the range 10^−16^–10^−4^ M. The slope of the straight line was S = 1.58 µA, with a correlation coefficient R^2^ of 0.99. The detection limit was calculated from the equation: Limit of Detection (LOD) = 3σ/S, where σ is the background for the blank. It is found to be 10^−16^ M. The relative standard deviation for three biosensors was 3.5%. The obtained detection limit was in the low range of those of the previously published laccase-based biosensors fabricated through the encapsulation of laccase in chitosan, as presented in Table 1 [9,10,11,12,13,14,15,16,17,18].

The repeatability of the biosensor was tested, the measurements were performed after storage of the biosensor at 4 °C. The relative standard deviation found after 15 days was 5%, showing the good preservation of the laccase in the chitosan+galactomannan composite.

### 3.4. Application of the Biosensor for the Compared Estimation of Phenolic Content in Olive Oils Samples

**Figure 6:**
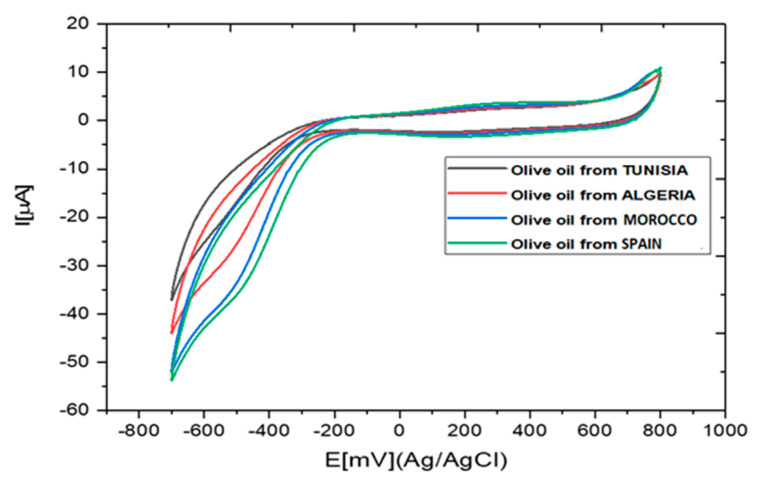
Cyclic voltammograms obtained in the presence of olive oil samples diluted in PBS (0.1 M, pH 7).

The cyclic voltammograms obtained in the extracts of olive oil samples diluted in 0.1 M PBS solution are presented in Figure 6. The cathodic peak potentials were in the same range as those obtained for the detection of catechol in 0.1 M PBS (Figure 5a).

**Figure 7:**
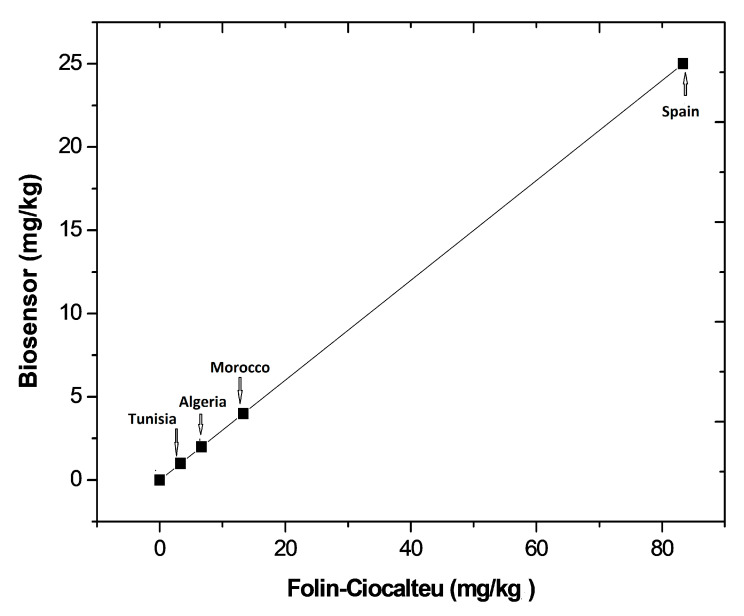
Graphical comparison of the results obtained with the laccase-based biosensor and with the Folin–Ciocalteu method.

A graphic comparison of the results obtained by the laccase-based biosensors and by the Folin–Ciocalteu method is shown in Figure 7. As can be observed, lower concentrations were systematically found with the biosensor compared to those obtained with the colorimetric method. This outcome could be explained considering the following: firstly, the Folin–Ciocalteu method estimates the total polyphenol content whereas the biosensor detects the concentration of pyrocatechol and other monophenols; moreover, the Folin–Ciocalteu reagent is considered a non-specific reagent by many authors since it can be reduced by non-phenolic compounds [25]. Thus, the Folin–Ciocalteu method could also reflect the presence of other oxidizable species present in the sample extract. Despite these differences, Figure 7 shows a high correlation between the results obtained by the two methods. The encapsulation of laccase in the chitosan+galactomannan composite prevents any inhibition effect of laccase by heavy metals and sulfhydryl compounds [26] that could be present in the olive oil samples. Accordingly, to estimate the antioxidant capacity of olive oil samples, we can conclude that the obtained laccase-based biosensor is a valuable alternative to the Folin–Ciocalteu method.

## 4. Conclusions

Galactomannan was extracted from carob seeds and characterized. It was used for the first time for the fabrication of a laccase-based biosensor by the encapsulation of laccase in a chitosan+galactomannan composite. This composite is able to ensure a good charge transfer rate. The laccase activity was well preserved in this composite and the sensitivity of detection of pyrocatechol in the 10^−16^–10^−4^ M range, was very high. The voltammetric response of the biosensor was stable for more than two weeks. To estimate the antioxidant capacity of olive oil samples, it was shown that the obtained laccase-based biosensor is a valuable alternative to the colorimetric Folin–Ciocalteu method.

## Figures and Tables

**Table 1:** Comparison of the analytical performance for previously published laccase biosensors using chitosan as immobilization matrix.

Laccase Immobilization Matrix	Linear Range (μM)	LOD (μM)	Shelf life Time (Days)	References
Laccase/MWCT/chitosan	0.091–12.1	0.233	-	[9]
LBL assemblies of chitosan/ionic liquid/phthalocyanine	2.4–26	8.96 × 10^−4^	-	[10]
Copper nanoparticles/chitosan/multiwalled carbon nanotubes/polyaniline-Au	1–500	0.156	10	[11]
Graphene/Chitosan Composite Film	2–100	0.26	10	[12]
Chitosan/Fe_3_O_4_ nanoparticles/reduced graphene oxide	6 × 10^−3^–0.228	18 × 10^−3^	60	[13]
Chitosan/AuNPs/Phthalocyanine	2.4–20	8.55 × 10^−4^	-	[14]
on Fe_3_O_4_/polyaniline/laccase/chitosan	0.5–80	0.4	60	[15]
chitosan modified with trymiristine	10^−14^–10^−9^	10^−14^	60	[16]
Chitosan- Lambda-Carrageenan	10^–14^–10^–8^	3 × 10^–15^	60	[17]
Graphene oxide -glycerol-chitosan	0.2–15	76 × 10^−3^	15	[18]
Chitosan/Galactomannan	10^–10^–100	10^–10^	15	This work

## Supplementary Materials

The following are available online at https://www.mdpi.com/2079-6374/10/6/70/s1, Figure S1: Molecular unit of galactomannan, Table S1: Redox peak maximum intensities (Ia and Ic) and Ep and for the different steps of the modification of the gold electrode.
